# Cardiac magnetic resonance left ventricular quantitative analysis post gadolinium: reliable and reproducible?

**DOI:** 10.1186/1532-429X-13-S1-P35

**Published:** 2011-02-02

**Authors:** Christopher J Rofe, Alison M Fletcher, David C Murday, Stephen P Harden, Charles R Peebles, James S Shambrook

**Affiliations:** 1 Southampton University NHS Hospitals Trust, Southampton, UK

## Objective

To assess the accuracy of left ventricular (LV) functional analysis derived from short axis steady-state free procession (SSFP) cine sequences following the administration of gadolinium contrast.

## Background

**Figure 1:**
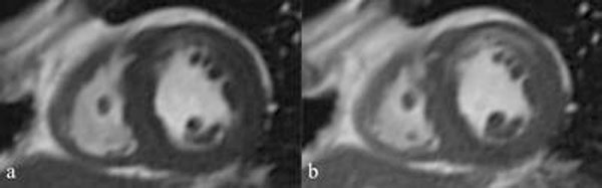
Pre (a) and post (b) gadolinium, SSFP end systolic short axis mid chamber views, in a patient with anterior wall subendocardial myocardial infarction. These images demonstrate the change in myocardial to blood pool contrast resolution, which could impact on the analysis.

Cardiovascular magnetic resonance (CMR) imaging is widely accepted as the gold standard in the assessment of LV volume and function. To date this assumption has been based on analysis of unenhanced gradient echo cine imaging. In certain clinical scenarios the use of gadolinium contrast is required for the evaluation of myocardial perfusion and late contrast enhancement. By performing an LV cine stack between the perfusion and late enhancement sequences the total scan duration can be reduced. However, it is possible that post contrast image acquisition alters endocardial and epicardial border detection and therefore affects the results of ventricular functional analysis (Fig. 1).

## Methods

13 patients, with a variety of clinical indications, undergoing cardiac MRI requiring gadolinium contrast enhancement had pre and post contrast SSFP short axis cine acquisitions obtained. All images were acquired using a Siemens 1.5T Avanto MRI scanner. The same radiographer acquired pre and post contrast short axis stacks using identical parameters and without patient repositioning.

Post processing was performed using Siemens Argus software for evaluation of left ventricular parameters including end diastolic volume (EDV), end systolic volume (ESV), stroke volume (SV), ejection fraction (EF) and mass. All data sets were analysed by three independent reporters.

Mean differences were compared using a 2-tailed paired t-test. Bland-Altman analysis was performed to assess inter-observer agreement. Values are quoted ± one standard deviation.

## Results

**Table 1:** Combined mean LV volume measurements by all observers for all subjects.

	Mean EDV (ml)	Mean ESV (ml)	Mean SV (ml)	Mean LV Mass (g)	Mean EF (%)
Pre Gd	178.7	96.2	82.5	129.1	51.0
Post Gd	182.8	97.3	85.7	126.6	51.5
p-value	0.083	0.604	0.129	0.426	0.671

Mean difference in EF pre and post gadolinium was 0.5±6.9%.

Bland-Altman analysis of inter-observer LV parameters demonstrates a mean difference in EDV pre gadolinium of 1.4±13.0 ml, post gadolinium of 2.9±13.3ml and in ESV pre gadolinium of 6.0±12.4 ml and post gadolinium of 1.5 ml±6.1.

## Conclusion

No significant difference between pre and post gadolinium assessment of LV parameters has been demonstrated. Despite the small sample size this suggests that use of post gadolinium data in LV analysis represents reasonable practice in a variety of patient groups.

